# Robot-assisted radical prostatectomy: Advancements in surgical technique and perioperative care

**DOI:** 10.3389/fsurg.2022.944561

**Published:** 2022-09-27

**Authors:** Isaac Palma-Zamora, Firas Abdollah, Craig Rogers, Wooju Jeong

**Affiliations:** Vattikuti Urology Institute, Henry Ford Hospital, Detroit, MI, United States

**Keywords:** Retzius-sparing, precision prostatectomy, single port surgery, robot-assisted radical prostatectomy (RARP), functional outcome, perioperative care

## Abstract

We reviewed the evolving strategies, practice patterns, and recent advancements aimed at improving the perioperative and surgical outcomes in patients undergoing robot-assisted radical prostatectomy for the management of localized prostate cancer.

## Introduction

Robot-assisted radical prostatectomy (RARP) using the DaVinci Surgical System (Intuitive Surgical, Sunnyvale, CA) was initially described at the turn of the century (1–3), and popularized by Dr. Mani Menon with the establishment of a robotic program at our home institution (4–6). The success of the robotic platform in the surgical management of prostate cancer enabled the dissemination of this technology within urology and other surgical fields. By 2015, RARP accounted for near 70%–85% of all radical prostatectomies performed in United States (US) (7–10). Since its initial approval by the US Food and Drug Administration (FDA) in 2000 for use in urological conditions, the DaVinci Surgical System has gone through several iterations (Standard, S, Si, Xi, X, and SP). Likewise, the RARP technique has been the subject of ongoing refinements aimed at improving surgical, oncologic, and functional outcomes. In this review, we described recent advancements in surgical technique and peri-operative care in patients undergoing RARP.

## Discussion

### Functional outcomes

Radical prostatectomy will invariably result in erectile dysfunction and urinary incontinence, which can negatively impact the quality of life of affected patients (11). The advent of RARP allowed for an intricate dissection of the prostate due to the improved and magnified vision of the robotic platform paving the way for many refinements in surgical technique. Despite these efforts, post-prostatectomy urinary incontinence has been reported anywhere between 1% and 69% of patients depending the definition and length of follow up (12, 13). Similarly, a report from a high-volume center suggested that post-prostatectomy potency rates have not significantly improved in the past 20 years despite a robotic approach and improvements in post-operative management including penile rehabilitation programs. Erectile function recovery rates were 27% and 34% at 12 and 24 months, respectively, and defined as ≥24 on a scale of 30 on a validated questionnaire, the International Index of Erectile Function 6 (IIEF-6) as reported by patients (14).

Return of urinary continence in the post-operative setting following radical prostatectomy is multifactorial. Structures believed to play a role, such as the endopelvic fascia, neurovascular bundle, puboprostatic ligaments, dorsal vascular complex, are all typically violated to some extent during the conventional RARP or open radical retropubic prostatectomy (ORRP). With this in mind, Bocciardi et al. described the Retzius-Sparing RARP (RS-RARP) in 2010 as a way to minimize iatrogenic urinary incontinence (15). Ensuing prospective studies by two different groups showed improved early urinary continence rates at 1 month as high as 92% (16, 17), which was corroborated by a randomized controlled trial (RCT) completed at our institution where RS-RARP was associated with higher continence rates at 1 month compared to the conventional anterior RARP (83% vs. 67%) (18). Improved early continence rates in RS-RARP have also been reported by other groups (19, 20); however, urinary control appears to be equivalent by 12 months (21, 22). Furthermore, RS-RARP limits iatrogenic damage to the bladder neck, which may have a synergistic effect in the early return of urinary continence as suggested by older studies in patients who underwent ORRP with bladder neck preservation (23–26). Additionally, RS-RASP was noted to have lower post-operative urinary dysfunction bother scores up to 1 month after catheter removal, suggesting an earlier return to baseline status compared to the anterior approach.

**Figure 1:**
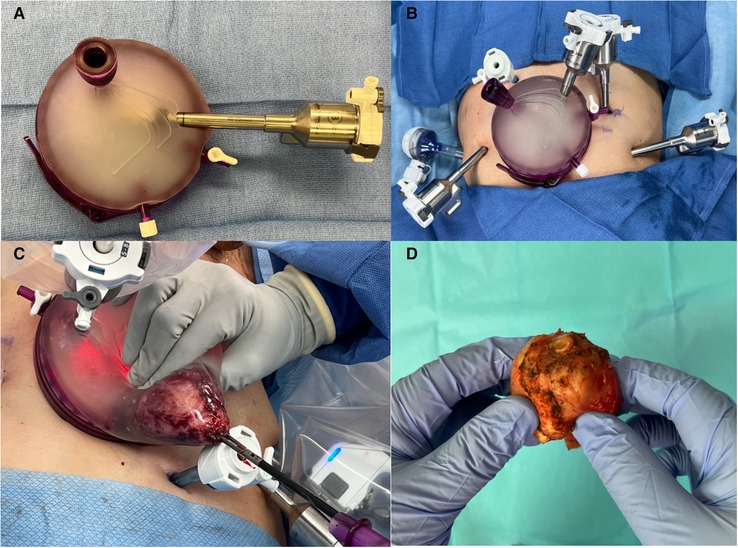
(**A**) GelPOINT access port for use in robot-assisted radical prostatectomy. For orientation purposes the insufflation port point to the left side of the patient, and the latch should point towards the feet when open. Robotic (camera port) and assistant trocars are pre-placed. (**B**) GelPOINT access port is placed *via* an infraumbilical 4 cm transverse incision. An additional five port incisions are made including a 12 and 5 mm assistant ports, both on the right side of the patient. For better triangulation during Retzius-Sparing robot-assisted radical prostatectomy, our preference is to medialize the most lateral left port site, and move the left paramedian port site 1–2 cm in the cephalad direction. (**C**) Specimen retrieval. A laparoscopic grasper is used to obtain a firm grip of the specimen through the urethra as it enters the apical prostate. The camera robotic arm is moved to an extracorporeal position within the GelPOINT and then lateralized to provide an unobstructed path for specimen retrieval through the assistant port. Note that the GelPOINT assistant port is usually extended sharply at time of placement in order to accommodate the retrieval of larger prostates. (**D**) Extracorporeal bimanual examination of prostate during robot-assisted radical prostatectomy.

Despite its improved continence control, adoption of the RS-RASP approach remains limited due to its technical difficulty and prolonged learning curve. An alternative is to perform Retzius space reconstruction at time of anterior RARP as some have reported improved continence rates (27). However, more studies are indicated to corroborate the efficacy of such technique. More recently, Retzius-Sparing using transvesical, extraperitoneal, and transperitoneal approach *via* a single-port robotic platform been described (28–30), but no direct comparison to the multi-port RS-RARP currently exists. Furthermore, RS-RARP is associated with questionable oncological control. A high rate of positive surgical margins (PSM) has been consistently reported on RS-RARP series (14%–28%) (16–19, 31, 32). A *post hoc* analysis of the RCT by Dalela et al. showed that the rates of biochemical recurrence free survival were comparable between RS-RARP and conventional RARP (22). In our practice, we routinely use a GelPOINT (Applied Medical, Rancho Santa Margarita, CA) access port when performing RARP (33). This is particularly useful during the Retzius-Sparing approach (1) as it allows intraoperative extracorporeal bimanual examination of the prostate to guide decision to obtain wider margins, (2) and provides greater intraoperative maneuverability of the camera port aiding the surgeon as the dissection approaches the prostatic apex and/or individuals with a deep pelvis (Figure 1). It should be noted that our institution has moved away from using regional pelvic hypothermia as internal review of our data revealed inconclusive results regarding post-operative functional outcomes. Added costs of the GelPOINT device should be considered ($550 at our institution). Furthermore, the potential need for a specimen retrieval bag remains ($36), which can be overcome *via* appropriate surgical planning as it relates to the size of midline skin/fascia incision relative to prostate size, and having an experienced bedside assistant that can successfully maneuver the extraction of the specimen.

Post-prostatectomy erectile dysfunction arises from the iatrogenic denervation of eretogenic nerves around the prostate including the neurovascular bundle (NVB) (34, 35). Initially pioneered by Dr. Patrick Walsh in the early 1980s (36), performing a nerve-sparing approach (NSA) became the standard of care in patients undergoing radical prostatectomy for localized prostate cancer if technically and oncologically feasible. Current perspectives on nerve-sparing are abound and generally fall into three categories: extent of dissection, minimizing iatrogenic nerve injuries, and adjuncts to guide dissection (37). Standard nerve-sparing aims at preserving each NVB. Patients with favorable oncological characteristics and mild to no erectile dysfunction may be candidates for an augmented nerve-sparing technique known as the “Veil of Aphrodite”, which is associated with improved potency rates following RARP (38–40). This technique preserves accessory nerves within the lateral prostatic fascia by developing an intra-fascial plane between the lateral pelvic fascia and the prostatic capsule. The dissection can be done in a retrograde fashion starting at the antero-apical aspect of the prostate and work towards the posterolateral base of the prostate, or vice versa (antegrade). The retrograde approach is associated with higher potency rates (41); while the antegrade approach may expediate the removal of prostate (40). A “Super Veil” technique has also been described, and involves sparing of the puboprostatic ligaments, which creates an avascular “hood” that preserves additional accessory nerves (42). Moreover, iatrogenic injury to erectogenic, or caversonal, nerves can arise from different sources. Excessive tissue traction may lead to ischemic injury (43). Additionally, judicious use of surgical clips and electrocautery should be advocated when dissecting close to the NVB and obtaining control of the prostatic pedicle as excessive usage could nullify the benefits of a nerve-sparing dissection. Furthermore, caversonal nerves are unmyelinated and particularly prone to thermal injury (37). A study from 2008 reported a 5-fold improvement in early return of sexual function following RARP with athermal nerve-sparing dissection (44). To this effect, a recent single-surgeon series reported on the use of clips vs. bipolar energy for control of the prostatic pedicle during RARP and found no difference in post-operative complications, and short-term functional and oncological outcomes between the two approaches (45). More recently, visual adjuncts have been developed that can potentially allow for a more intricate dissection of the prostate (37). The use of indocyanine green (ICG) may help in the surgical preservation of pertinent blood vessels as it can help identify the prostatic artery and other accessory arterial branches that may play role in maintaining erectogenic function. On the other hand, multi-parametric magnetic resonance imaging (MRI) of the prostate improves patient selection as it helps guide the extent, or lack thereof, of nerve-sparing techniques in up to 47% of patients undergoing RARP (46). Similarly, augmented reality (AR) is a novel technology that has shown promise in patients undergoing RARP (47). Its role is analogous to that of a prostate MRI fusion biopsy where a three-dimensional rendering of the prostate is obtained *via* MRI and fused with the live feed on the robotic console, which allows the surgeon to adjust its dissection plane in real-time.

Despite improved surgical techniques, concerns for urinary incontinence and erectile dysfunction persist both in patients and provider, prompting many to pursue alternative management strategies such as active surveillance and focal therapy that are associated with a more favorable side-effect profile. Recently, Dr. Mani Menon developed the Precision Prostatectomy (MPP), a new organ-preserving surgical approach for the management of low to intermediate-risk prostate cancer (48, 49). The MPP involves subtotal resection of the prostate with a radical excision on the side of the index lesion while a thin 5–10 mm rim of tissue, including the prostatic capsule and seminal vesicle, is deliberately preserved on the contralateral side with less cancer burden. This procedure is intended for patients with favorable risk prostate cancer who are pre-operatively potent, and are willing to follow an active surveillance protocol post-operatively. Early results are promising with 85% of all-comers and 90% of the pre-operatively potent men being potent at 12 months, with rates of residual cancer and need for secondary procedures appear to be equivalent or superior to those of who undergo high-intensity focused ultrasound (HIFU) (48). The precision prostatectomy offers a favorable cancer control compared to less invasive focal therapy techniques, and minimizes the risk of overtreatment associated with whole gland treatments such as radiation or radical prostatectomy.

### Post-operative care

Post-operative bladder drainage in patients who undergo RARP can be achieved *via* urethral or suprapubic catheter (SPC). Our institutional SPC technique was initially described in 2009 and associated with less patient discomfort (50). Overall, there was a 4.4% rate of complications attributable to SPC with most taking place in the immediate post-operative period and managed conservatively or with the conversion to urethral catheter. In rare occasions, patient required need for prolonged catheterization (<0.6%) or had formation of bladder neck contracture (<0.3%) (51, 52). These findings have been corroborated by other high-volume centers and a recent meta-analysis (53–55). To this date, SPC remains a safe and viable option for interested patients. Currently, we use a 14F Ultrathane® with Mac-Loc (Cook Medical, Bloomington, IN) SPC that is typically removed at seven days without the need of a urethral catheter at any point in the post-operative setting.

Pain management in the post-operative setting is of the utmost importance. We employed a multimodal approach that minimizes opioid analgesics. At the conclusion of each case, and when not clinically contraindicated, patients receive incisional local anesthetic installation, insertion of a belladonna and opium suppository, and administration of intravenous (IV) ketorolac. Post-operatively, patients remain on staggered scheduled ketorolac and a combination of acetaminophen and methocarbamol. On discharge, patients receive a pro re nata 10-day supply of acetaminophen, ibuprofen, and methocarbamol. Our approach is similar to that of recently published studies reporting on the excellent analgesic effect of opioid-sparing protocols following RARP with the exception that we do not use pre-operative rehabilitation pathways (56, 57). The caveat is that post-surgical pain-control in patients after a radical prostatectomy tends to be favorable regardless of approach. A prospective study from 2005 in patients undergoing RARP vs. open-radical prostatectomy found that pain scores on POD 1 were no different between the two groups (58).

There has been a shift towards same-day discharge (SDD) for patients undergoing RARP. First reported in 2007, a select group of 11 patients had favorable outcomes following extraperitoneal RARP and able to be discharged home the same day (59). Since then, multiple groups have corroborated the safety and feasibility of SDD-RARP; however, study sample sizes were generally small until recently (60–62). Large single and multi-centered studies in patient undergoing RARP have reported favorable results following SDD (63, 64). The commonality amongst these studies is the importance of established protocols that promote the multidisciplinary collaboration between the surgeon, anesthesia team, and nursing staff to ensure a safe discharge to home. Predictors of successful SDD include being first or second case of the day (61, 63, 64), which allows for an extended period of monitoring prior to discharge later in the day. Recently, single (SP) and multiport (MP) robotic approaches have been described in patients undergoing SDD RARP with rate of SDD higher in those undergoing SP procedures (65). Furthermore, it appears that minimizing iatrogenic peritoneal irritation either from an underlying pneumoperitoneum, transperitoneal incisions, and mobilization of bladder or peritoneal contents, maximizes chances of successful SDD following RARP. This can be achieved by employing ultra-low pneumoperitoneum of 6 mm Hg as commonly done by Abaza et al. using either SP and MP robotic platforms (63, 65, 66), or by employing alternative methods and performing RARP *via* extraperitoneal (88% SDD) (67) or transvesical (65% SDD) (29) approaches *via* the SP robotic platform. On the contrary, there is disagreement if a pelvic lymph node dissection impairs chances of SDD but it appears to be less of an issue as experience with SDD grows (63, 64, 68). At our institution, SDD-RASP is routinely performed on well-motivated patients using the SP or MP robotic platform with extraperitoneal or transperitoneal approaches available depending on surgeon's preference and patient factors.

### Single-port surgery

The DaVinci SP Surgical System (Intuitive Surgical, Sunnyvale, CA) is the latest and most advanced iteration of the robotic surgical platform commonly used to perform urological procedures (SP Robot). Single-port prostatectomy was initially described in 2008 in patients with large-volume benign prostatic hyperplasia (BPH) who underwent laparoscopic transvesical enucleation of the prostate, which was followed by a small series reporting on the feasibility of single-port laparoscopic radical prostatectomy by the same group (69, 70). In the ensuing decade, multiple groups described the use of premarketing versions of the SP Robot (SP999 and SP1098) in the successful completion of RARP and other urological procedures in both clinical and pre-clinical settings (71, 72). This should not be confused with the DaVinci Single Site (Intuitive Surgical, Sunnyvale, CA) technology that uses a single incision, a GelPOINT or its equivalent, and utilizes curved instruments that are compatible with DaVinci MP robotic platforms. Single-site approaches for RARP and other urological procedures have been described; however, its relevance remains uncertain but may be an option where the SP robotic platform is not available (73–76).

Usage of the SP Robot involves an “adjusting” curve as most surgeons transitioning to the SP platform likely have significant experience performing MP-RARP. The Endowrist® technology in the SP platform maintains 7 degrees of freedom albeit using a different mechanism, which can dramatically change the dynamics of operating in confined spaces. Furthermore, the reduced footprint of the SP limits the ability of a bedside assistant to aid the surgeon with retraction and suction throughout the procedure. Multiple approaches have been developed to circumvent around these limitations. The remotely operated suction irrigation (ROSI) system is surgeon-controlled and minimizes reliance on a bedside assistant for suctioning in patients undergoing SP or MP surgery including RARP (77). The use of a non-invasive magnetic retraction device (Levita™ Magentic Surgical System, San Mateo, CA) has been described in RARP using both SP and reduced-port approaches (78, 79). Furthermore, not all SP-RARP are truly single-port as in some instances an additional port may be placed at the surgeon's discretion, an approach commonly known as the SP + 1. Both pure SP (28, 65, 80) and SP + 1 (67, 79, 81–83) approaches have been reported by prominent groups. In our experience, the SP + 1 was commonly used early in our experience with the SP robotic platform, but now it is reserved for complex cases where dynamic bedside assistance may be warranted.

Following FDA approval of the SP Robot in 2018 for use in urological procedures, a multitude of groups across the globe have reported their initial experience performing RARP with the SP robot (65, 67, 79, 83, 84). Not surprisingly, various approaches and techniques have emerged. The conventional transperitoneal RARP is the most commonly done approach using the SP platform and it usually involves the use of an additional port incision for bedside assistance (67, 79, 81–83, 85–87), albeit the largest series of SP-RARP outcomes was performed using a pure single-port transperitoneal approach (63). Second most common SP-RARP modality is *via* an extraperitoneal approach (87), which was described using pure SP that allows for SDD (28), SP + 1 with a drain that is removed on POD 1 (88), and single-site using a MP robotic platform (74, 76). The extraperitoneal approach may be indicated if planning for SDD as it minimizes peritoneal irritation, or for men with hostile abdomen and/or comorbidities at odds with maintaining a prolonged pneumoperitoneum (28, 88). Transperitoneal Retzius-sparing SP-RARP has been reported with varying degrees of success, which was defined as avoiding conversion to an anterior approach (80, 81). Other lesser known SP-RARP approaches include the transperineal and transvesical approaches, as previously described by Dr. Jihad Kaouk et al. A transperineal SP-RARP was described in 26 patients with relative contraindications for a retropubic approach and noted to be technically challenging; however, it was associated with higher rates of positive surgical margins (23% vs. 65%) and comparable functional and oncological outcomes to those of the conventional MP-RARP at 12 months (89). A purely transvesical SP-RARP with limited pelvic lymph node dissection was initially described as an option for patients with low risk of lymph node metastases and a “Frozen Pelvis” that limits access whether it be transperitoneal or extraperitoneal, and where the intravesical lumen provides a big-enough space for maneuvering needed to perform a SP-RARP (29). Advantages include the Retzius-sparing nature of this approach and associated improved urinary control along with avoidance of peritoneal contents and a theoretical lower risk of complications that make this approach and attractive option in all patients regardless of history of prior abdominal surgery (29). To date, the use of SP-RARP offers similar outcomes compared to MP-RARP in terms of urinary control and erectile function; longer follow-up is needed to determine if they are oncologically equivalent (87).

## Conclusions

Robot-assisted radical prostatectomy is a safe procedure that can be performed in a myriad of ways using the SP or MP robotic platforms. Specific techniques and approaches will vary depending on the surgeons preference, patient expectations, clinical factors, and tumor characteristics. Nonetheless, the goal should be to obtain oncological control, maintain sexual potency, and minimize urinary incontinence.

## Author contributions

IP-Z — manuscript. FA — review, revision. CR — review, revision. WJ — manuscript, review, revision. All authors contributed to the article and approved the submitted version.

## Conflict of interest

The authors declare that the research was conducted in the absence of any commercial or financial relationships that could be construed as a potential conflict of interest.
